# Efficacy of Imidazolium Salt in Disinfecting Soft Contact Lenses Contaminated with *Acanthamoeba* spp. Trophozoites

**DOI:** 10.1007/s11686-026-01249-6

**Published:** 2026-03-12

**Authors:** Natasha Cristine Gonçalves, Igor Luiz Gonçalves Pereira, Henri Stephan Schrekker, Diane Ruschel Marinho, Marilise Brittes Rott

**Affiliations:** 1 Protozoology Laboratory, Microbiology Immunology and Parasitology Department, Basic Health Sciences Institute, Federal University of Rio Grande do Sul, 2600 Ramiro Barcelos Street, Porto Alegre, RS 90035-002 Brazil; 2 Laboratory of Technological Processes and Catalysis, Institute of Chemistry, Federal University of Rio Grande do Sul, 9500 Bento Gonçalves Avenue, Porto Alegre, RS 91501-970 Brazil; 3 Cornea Department, Ophthalmology Service, Porto Alegre Clinical Hospital, 2350 Ramiro Barcelos Street, Porto Alegre, RS 90035-903 Brazil

**Keywords:** Acanthamoeba, Contact lenses, Keratitis, Imidazolium salt, Cornea

## Abstract

**Objectives:**

*Acanthamoeba* spp. can infect the cornea due poor lens hygiene allows colonization, and current cleaning solutions are ineffective due to resistant cyst formation. This study aimed to establish, for the first time, a standardized protocol for *Acanthamoeba* trophozoite adhesion to silicone hydrogel soft contact lenses and to quantify the impact of lens–drug interactions on antiparasitic efficacy. Additionally, the study evaluated the efficacy of 1-hexadecyl-3-methylimidazolium chloride (C_16_MImCl) against *Acanthamoeba* trophozoites in the presence of soft contact lenses, in comparison with a commercial contact lens solution and chlorhexidine.

**Methods:**

A protocol for trophozoites adhesion to silicone hydrogel contact lenses was developed, and the antiparasitic activity of C_16_MImCl was compared to that of a commercial solution and chlorhexidine.

**Results:**

C_16_MImCl effectively eliminated all trophozoites in the presence of contact lenses and prevented encystment at concentrations ten times the minimum inhibitory concentration, despite partial absorption by contact lenses. Notably, it showed greater corneal cell viability than chlorhexidine, highlighting its potential as a safer alternative.

**Conclusion:**

These results underscore its strong anti-amoebic activity, outperforming the commercial solution tested. While further cytotoxicity studies and tests simulating ocular conditions are warranted, C_16_MImCl emerges as a promising candidate for a future contact lens cleaning and decontamination solution.

## Introduction

Free-living amoebae (FLA) are single-celled protozoa found in diverse environments, including water, soil, and air [1]. Among these, the genus *Acanthamoeba* is particularly notable due to its ability to cause severe human diseases, such as amoebic keratitis (AK) and granulomatous amoebic encephalitis (GAE) [2].

Amoebic keratitis primarily affects healthy individuals, especially contact lens (CL) users. Although AK remains relatively rare, its incidence has increased in recent years, largely due to the growing use of CL. Improper lens hygiene and care, including exposure to contaminated water, are key risk factors, as CL and their cases can serve as sources of *Acanthamoeba* contamination [1]. Additionally, the widespread availability of CL-sold over-the-counter and online-has facilitated unsupervised use, often without proper prescriptions or guidance [3, 4]. The development of AK can also occur in individuals who are not CL users due to injuries and exposure to contaminated soil and water [5].

*Acanthamoeba* exists in two morphological forms: trophozoites and cysts [6]. The trophozoite is the active, infective stage capable of penetrating the corneal epithelium through micro-abrasions caused by CL debris or some mechanical injury [1, 7]. In contrast, the cyst is a highly resistant, dormant form, capable of surviving harsh environmental conditions for up to 20 years [8]. Its double-layered wall offers protection against changes in pH, humidity, temperature, and nutrient availability [9].

Clinically, AK manifests as a painful, progressive corneal infection characterized by redness, inflammation, and gradual vision loss, potentially resulting in blindness [9]. The infection typically occurs when CL come into contact with water contaminated with *Acanthamoeba* spp. The amoeba invades the corneal tissue through epithelial abrasions, whether caused by incorrect CL use or accidental injury. The pathogenic process involves phagocytosis, cytolysis, apoptosis, and a robust inflammatory response, ultimately leading to stromal degradation and visual impairment [10].

Furthermore, the genus *Acanthamoeba* is known as the “Trojan horse of the microbial world” [11], as it has the ability to carry microorganisms within itself, either through predation (phagocytosis) or even to facilitate gene exchange and increase the virulence of these microorganisms [12]. The intracellular presence of *Pseudomonas aeruginosa* in *Acanthamoeba* spp. has been demonstrated, and this can lead to coinfection, increasing the severity of keratitis. The presence of organic material and bacteria in an environment promotes amoebal growth, as they serve as food for this protozoan. Some bacteria are able to survive this process [13], enabling the exchange of genetic material between these microorganisms. Therefore, a significant proportion of *Acanthamoeba* spp. isolates harbor endosymbionts within their cells [14]. Identifying the presence of endosymbionts is relevant because, depending on the intracellular microorganisms they harbor, it may be necessary to employ differentiated therapeutic strategies, considering potential impacts on drug resistance and responses to antimicrobial agents.

Despite growing awareness, the treatment of AK remains challenging, particularly due to the organism’s highly resistant cyst form [15]. Currently, there are no specific medications targeting *Acanthamoeba* spp., and existing therapies are often lengthy and complex, requiring the hourly application of topical agents in the initial phase [16]. Additionally, some drugs can be toxic to the cornea, leading to complications such as ulceration [17].

Drugs such as biguanides (chlorhexidine and polyhexamethylene biguanide), aromatic diamidines, aminoglycosides, and imidazoles are used against AK [18]. The trophozoites are more sensitive to the action of these drugs; however, due to cyst resistance, in many cases it is necessary to use combined therapies, such as topical biguanides with diamidines in the initial phase of treatment [19].

Due to its ubiquity—especially in aquatic environments—*Acanthamoeba* can be encountered during common activities such as swimming in pools, lakes, oceans, or even showering while wearing CL [2]. Risk factors for AK include improper handling of CL, such as using tap water or saline solution for cleaning, poor hand hygiene, sleeping with lenses, using expired products, and exposure to biofilms formed by other microorganisms. These practices facilitate amoebic adhesion and infection [7].

CL are manufactured from a variety of materials, including hydrogel and silicone hydrogel. Silicone hydrogel lenses, widely used due to their high oxygen permeability and comfort, are composed of hydrophilic and hydrophobic monomers [20]. These properties promote lens hydration, oxygen exchange, and wearer comfort but may also contribute to microbial adhesion due to their irregular surface and increased wettability [21].

Each manufacturer develops proprietary silicone hydrogel formulations. The lenses used in this study—Comfilcon A Biofinity^®^ (52%)—are monthly-use, hydrophilic lenses containing 48% water. These CL are indicated for the correction of refractive errors such as myopia, hyperopia, astigmatism, and presbyopia. Their design allows for high oxygen transmissibility and moisture retention, which may, paradoxically, facilitate microbial colonization [22, 23].

Poor lens hygiene can lead to biofilm formation and ocular infections, including AK. The hydrated, irregular surface of silicone hydrogel lenses may promote the adhesion of *Acanthamoeba* spp [21]. Additionally, micro-abrasions from improper use—such as prolonged wear, sleeping with lenses, or contamination with debris—can compromise the corneal epithelium and serve as entry points for pathogens [7].

Commercial lens cleaning solutions typically contain antimicrobial agents, surfactants, buffers, and chelators [24]. However, these solutions often lack efficacy against *Acanthamoeba* cysts due to the robust double-layered wall composed of proteins, lipids, and carbohydrates [25, 26], but, mainly, this resistance to biocides is related to the presence of cellulose in its wall [27]. Some multipurpose solutions may even induce encystment, further complicating treatment [28]. Therefore, the development of more effective disinfecting solutions is urgently needed.

One promising class of compounds is imidazolium salts (IS), which possess antibacterial, antifungal, anti-inflammatory, and antiparasitic properties [29–34]. These organic compounds, composed of tunable cations and anions, are characterized by high thermal stability, ionic conductivity, and the ability to dissolve both organic and inorganic compounds. IS are already in use in chemical, food, pharmaceutical, and biotechnological industries [31].

Notably, Fabres et al. [34] demonstrated that the IS 1-hexadecyl-3-methylimidazolium (C_16_MImCl) exhibits activity against *Acanthamoeba* trophozoites. Further studies by Dos Santos and Santos et al. [35, 36] established the 24-hour minimum inhibitory concentrations (MIC) of C_16_MImCl against both trophozoites (15.62 µg/mL for isolated MZ404337 and 7.81 µg/mL for isolated MZ404332) and cysts, supporting its potential as an anti-*Acanthamoeba* agent. While its exact mechanism of action remains unclear, it is believed to involve interactions with cellular and mitochondrial membranes [34, 37]. These findings support the potential development of a novel lens cleaning and disinfection solution based on C_16_MImCl, which had not previously been assessed for CL applications up to that point.

We hypothesized that silicone-hydrogel CL reduce the bioavailability of C_16_MImCl due to material–drug interactions, requiring higher effective concentrations to inactivate adhered trophozoites. Herein, we report the first study to (i) evaluate C_16_MImCl activity on trophozoites in the presence of CL, (ii) demonstrate that probable absorption by silicone hydrogel reduces MIC efficacy, and (iii) report a standardized adhesion protocol for soft lenses.

## Materials and Methods

### Selection of *Acanthamoeba* Samples

Two clinical isolates of *Acanthamoeba* spp., classified as genotype T4, obtained from corneal scrapings of patients diagnosed with AK at the Hospital de Clínicas de Porto Alegre, were used in this study. The isolates are registered in GenBank under accession numbers MZ404337 and MZ404332. Notably, the latter harbors an internalized endosymbiont, *Candidatus Paracaedibacter acanthamoebae* [38].

### Trophozoite Culture

Axenic cultures of *Acanthamoeba* trophozoites were maintained in cell culture flasks containing PYG medium (2% proteose peptone, 0.2% yeast extract, 1.8% glucose) supplemented with penicillin (10,000 IU/mL) and streptomycin (10 mg/mL), incubated at 30 °C [9]. After incubation, trophozoites were harvested by centrifugation at 500 rpm for 5 min. The resulting pellets were resuspended in fresh PYG medium and adjusted to a final concentration of 10⁴ cells/mL.

### Imidazolium Salt C_16_MImCl

The IS C_16_MImCl was synthesized following the method described in the literature [36, 39], and its ^1^H NMR spectrum agrees with the reported data. Dried C_16_MImCl was diluted in Milli-Q^®^ water to reach the desired concentrations.

### Commercial Multipurpose Solution

For comparative efficacy analysis, a commercial cleaning and disinfection solution for soft (gelatinous) CL was tested against *Acanthamoeba* spp. trophozoites. The formulation contains hydroxyalkylphosphonate, boric acid, disodium edetate, poloxamine, sodium borate, sodium chloride, and 0.0001% polyaminopropyl biguanide.

### Contact Lenses

This study used soft CL composed of 52% Comfilcon A silicone hydrogel and 48% water. As a third-generation silicone hydrogel material, Comfilcon A offers high oxygen transmissibility (Dk/T), promoting increased oxygen flow to the cornea. The lenses incorporate patented Aquaform^®^ technology, which enhances water retention and maintains lens hydration. They are disposable, designed for the correction of myopia, and were supplied by Dr. Diane Ruschel Marinho of the Departament of Ophtalmology at the Hospital de Clínicas de Porto Alegre.

### Evaluation of the MIC Activity of C_16_MImCl on *Acanthamoeba* spp. Trophozoites

To verify a possible interaction between CL and C_16_MImCl, an evaluation of the MIC activity of C_16_MImCl in 24 h without CL interference was performed only for isolate MZ404332, using two different C_16_MImCl batches. In a 96-well plate, 10⁴ trophozoites/mL were seeded and C_16_MImCl at its MIC (7.81 µg/mL). After homogenization, the plate was incubated for 24 h at 30 °C. Then, the viability of *Acanthamoeba* spp. trophozoites was visualized using 0.4% Trypan blue dye, which penetrates the membrane of damaged cells (a dark blue color indicates nonviability). Subsequently, 40 µL of this suspension was plated on NNA (1.5% Non-Nutrient Agar) plates seeded with heat-inactivated *Escherichia coli*. These plates were incubated at 30 °C and kept in an oven for 15 days for observation. These experiments were performed in triplicate, with one replicate for each C_16_MImCl batch. The batches refer to samples of C_16_MImCl acquired in 2023 and 2025.

### Evaluation of C_16_MImCl Activity at Its MIC and at 5× and 10× MIC Concentrations

Initially, the trophozoite adhesion protocol to the CL would be performed with C_16_MImCl at its MIC (15.62 µg/mL for MZ404337 and 7.81 µg/mL for MZ404332). However, after conducting preliminary experiments using the MIC, it was decided to perform a screening test using the MZ404337 isolate, aiming at time efficiency, with C_16_MImCl at concentrations five and ten times higher than the MIC. This experiment was performed using the same methodology as the adhesion test (described in the next Sect. 2.8): 10⁴ trophozoites/mL were added to a 24-well plate, along with C_16_MImCl at concentrations of 78.1 µg/mL (5X above the MIC) and 156.2 µg/mL (10X above the MIC), PYG and the already cut CL were added. After homogenizing with a pipette, the plate was shaken at 40 rpm at 30 °C for 90 min. Then, the plate was incubated without shaking in an oven for 24 h at 30 °C. After 24 h, CLs were washed with phosphate-buffered saline (PBS) and observed under a microscope at 100x and 400x magnifications. Immediately afterward, CLs were incubated at 30 °C on NNA plates seeded with heat-inactivated *E. coli* and kept (for observation) in the bacteriological incubator for 16 days. Given that this experiment served as a preliminary screening, tests were carried out in duplicate with a single repetition.

### Protocol for assessing *Acanthamoeba* spp. Trophozoites Adhered to Contact Lenses and the Antiamoebic Activity of C_16_MImCl

The effect of C_16_MImCl on the adhesion of *Acanthamoeba* trophozoites to CL was evaluated using a protocol adapted from the methodology described by Pinto [17] for *Acanthamoeba* adhesion to scleral lenses. A standardized adhesion protocol was developed for silicone hydrogel soft CL. After establishing the C_16_MImCl concentration (as described in item 2.7), in a 24-well flat-bottom plate (Kasvi^®^), *Acanthamoeba* trophozoites were seeded at a concentration of 10^4^ cells/mL in PYG medium. C_16_MImCl was added at concentrations of 156.2 µg/mL for isolate MZ404337 and 78.1 µg/mL for isolate MZ404332 (both concentrations 10 times higher than MIC determined in the studies Dos Santos and Santos et al.) [35, 36]. Silicone hydrogel CLs were included in each well, bringing the final volume to 2 mL per well. Due to their curvature, the CLs were trimmed along the edges to facilitate microscopic observation. Using tweezers and scissors disinfected with 70% ethanol, each lens was carefully cut three times at the periphery to allow the entire surface to be visualized under the microscope. For controls, 0.02% chlorhexidine was used as a positive control, and negative control consisted of trophozoites, PYG medium, and CL without treatment. Additionally, trophozoites at the same concentration were centrifuged, and only the pellet was added to the wells containing 2 mL of the commercial solution, without dilution, exactly as specified by the manufacturer, simulating the real conditions of CL use.

To determine the residual concentration of C_16_MImCl in the assay medium after contact with the CL, 1 mL aliquots of the solution were collected from the wells following lens removal. Samples were diluted 1:1 (v/v) with diluent and analyzed by HPLC using a Phenomex C18 column (150 × 4.6 mm, 5.0 μm) with a C18 guard cartridge (SecurityGuard). The mobile phase consisted of acetonitrile/phosphate buffer pH 4 (80:20, v/v) under isocratic conditions, with a flow rate of 1.0 mL/min, column temperature of 35 °C, and detection at 216 nm. The diluent used for all samples was acetonitrile/phosphate buffer pH 4 (60:40, v/v). The total run time for each analysis was 7 min.

The plates were incubated at 30 °C on an orbital shaker at 40 rpm for 90 min to promote trophozoite adhesion to the CL surfaces. Following this, the plates were transferred to a bacteriological incubator and maintained at 30 °C for a total of 24 h (including the initial 90 min). After incubation, the CL were transferred using sterile tweezers to new wells containing 2 mL of non-ice-cold 1x PBS to halt the action of C_16_MImCl. The plates were again incubated on an orbital shaker at 40 rpm for 1 min at 30 °C to wash the lenses and remove non-adherent trophozoites.

**Fig. 1:**
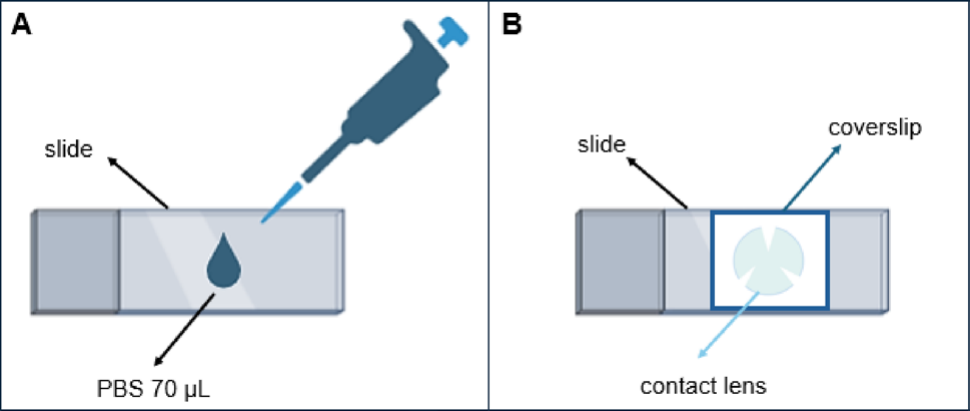
Illustration of the slide preparation process for microscopic visualization of *Acanthamoeba* trophozoites adhered to contact lenses

For microscopic analysis, 70 µL of PBS was placed on a microscope slide, and each lens was positioned on this droplet. A coverslip was gently placed on top of the lens (Fig. 1). PBS was applied to minimize air bubbles and allow full visualization of the lens surface. The lenses were examined under a light microscope using 10x and 40x objectives to assess trophozoite adhesion across the entire lens area. To confirm the presence of viable trophozoites, the CL were inoculated onto NNA plates previously seeded with heat-inactivated *E. coli* and incubated at 30 °C for up to 15 days.

### HCE-T Corneal Cell Culture

Human corneal epithelial cells (HCE-T) used in this study were provided by Dr. Araki Sasaki and the RIKEN Cell Bank, Japan. Cells were cultured in filtered Dulbecco’s modified Eagle’s medium/nutrient mixture F-12 (DMEM/F12, Gibco), supplemented with calcium bicarbonate, 10 ng/mL human epidermal growth factor (hEGF), 5 µg/mL recombinant insulin, 5% fetal bovine serum (FBS), and antibiotics (10,000 IU/mL penicillin and 10 mg/mL streptomycin). Cultures were maintained at 37 °C in a humidified atmosphere with 5% CO₂. Upon reaching 80–90% confluence, adherent cells were detached using trypsin and used in subsequent experiments.

### HCE-T Viability Assay

To assess the cytotoxicity of C_16_MImCl on corneal cells, a resazurin assay was performed. Resazurin is a cell-permeable redox dye that is reduced by metabolically active (viable) cells from its native blue, non-fluorescent form into resorufin, a pink, fluorescent compound. Thus, the amount of resorufin generated is directly proportional to the number of viable cells and can be quantified by either fluorescence or absorbance [40, 41]. In this study, cell viability was quantified via fluorescence.

The assay protocol was adapted from Riss et al. and Eilemberger et al. [40, 42]. Resazurin powder was diluted in PBS to a stock concentration of 0.15 mg/mL and filtered through a 0.2 μm membrane in the dark. The prepared stock solution was stored refrigerated until use. HCE-T cells were seeded in 96-well plates at a density of 10⁴ cells per well in a final volume of 100 µL per well. The plates were incubated for 24 h at 37 °C in a humidified atmosphere containing 5% CO₂ to allow for cell adhesion. After incubation, the culture medium was removed, and 100 µL of C_16_MImCl was added at concentrations of 1.86, 3.71, 7.44, 14.88, 29.76, and 59.52 µg/mL. The subsequent steps were performed in the dark to ensure assay accuracy. Following a 2-hour incubation with C_16_MImCl, the medium was discarded, and resazurin solution was added to each well. The plate was then wrapped in aluminum foil and incubated for an additional 2 h. Fluorescence was measured using a microplate reader (excitation: 560 nm; emission: 590 nm).

For experiments involving a 4-hour exposure to C_16_MImCl, the same procedure was followed, except that resazurin was added directly to the wells without removing the medium, ensuring continuous exposure during the entire 4-hour period. Chlorhexidine (0.02%; 200 µg/mL) was used as a positive control. Wells containing only cells and resazurin served as the negative control. Each condition was tested in quadruplicate with two independent replicates. Two blank wells containing medium and resazurin (without cells) were included to account for background fluorescence. Results are expressed as the percentage of cell viability relative to the negative control and presented as mean ± standard deviation (SD). Statistical analysis was performed using GraphPad Prism 10 (GraphPad Software, San Diego, CA, USA). Group means were compared using one-way ANOVA, and statistical significance was set at *p* < 0.0001.

###  Declaration of Generative AI and AI-Assisted Technologies in the Writing Process

During the preparation of this work, the authors used ChatGPT (OpenAI) to assist with language refinement and writing improvement. All content was subsequently reviewed and edited by the authors, who take full responsibility for the final version of the manuscript.

## Results

### Evaluation of the MIC Activity of C_16_MImCl on *Acanthamoeba* spp. Trophozoites

The test results to verify the 24-hour MIC activity of the C_16_MImCl for isolate MZ404332 without the influence of CL through viability using Trypan blue dye (0.4%) and the confirmation of these results can be analyzed in Table 1. The C_16_MImCl batches correspond to the C_16_MImCl obtained in 2023 and 2025. In both tests, both C_16_MImCl inactivated 100% of the trophozoites. Therefore, the efficacy of the MIC (7.81 µg/mL) previously determined in a study by the group [31] was confirmed, and there was no difference in the C_16_MImCl activity of different batches.

**Table 1:** MIC assessment test for MZ404332 without CLs

	Trypan blue cell viability		NNA plates	
	Batch 2023	Batch 2025	Batch 2023	Batch 2025
A	100% non-viable	100% non-viable	100% non-viable	100% non-viable
B	100% non-viable	100% non-viable	100% non-viable	100% non-viable
C	100% non-viable	100% non-viable	100% non-viable	100% non-viable

MIC: Minimum Inhibitory Concentration; CLs: contact lenses. A, B, and C: technical replicates

### Evaluation of C_16_MImCl Activity at Its MIC and at 5× and 10× MIC Concentrations

Regarding the analysis of concentrations 5 and 10 times higher than the MIC of C_16_MImCl against trophozoites, the following results were obtained. After 24 h of incubation of the CLs with C_16_MImCl at a concentration 5 times higher (78.1 µg/mL) than the MIC, sensitized trophozoites — both morphologically altered and normal — were observed under light microscopy. This was confirmed, as trophozoite growth was observed on the NNA plates after 16 days of incubation.

At a concentration 10 times higher (156.2 µg/mL) than the MIC, 100% of the trophozoites were identified as undergoing cell lysis, indicated by the loss of plasma membrane integrity, as observed under light microscopy. This result was confirmed, as no trophozoite growth was observed on the NNA plates after 16 days.

However, when MIC was applied, it was observed that the trophozoites remained viable and their growth was observed on the NNA plates (data not shown).

Therefore, the C_16_MImCl solution at 10 times the MIC was selected for trophozoite adhesion tests on CLs — specifically, 156.2 µg/mL of C_16_MImCl for MZ404337 and 78.1 µg/mL for MZ404332.

### Antiamoebic Activity of C_16_MImCl in Preventing the Adhesion of *Acanthamoeba* spp. Trophozoites to Contact Lenses

**Fig. 2:**
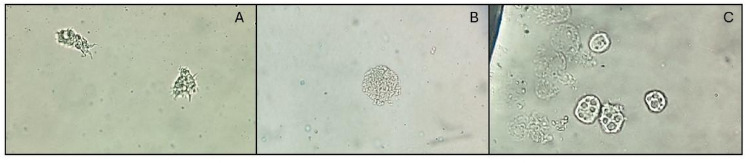
Optical micrographs at 400x magnification of (A) intact *Acanthamoeba* spp. trophozoites adhered to a contact lens (negative control); (B) trophozoites with ruptured membranes and cytoplasmic granule release, indicating lysis (with treatment chlorhexidine); and (C) trophozoites with altered morphology, lacking acanthopodia and appearing rounded and irregular, consistent with early-stage functional impairment (when exposed to the multipurpose solution)

The results of the antiamoebic activity of C_16_MImCl in the adhesion tests for isolates MZ404337 and MZ404332 are presented in Table 2. Trophozoites exhibiting altered morphological structures were observed, including ruptured plasma membranes accompanied by cytoplasmic granule release both within the cell and in the surrounding area, indicating cell lysis (Fig. 2A vs. Fig. 2B). In such cases, the affected trophozoites were not considered to be adhered to the CL surface. Additionally, some trophozoites lost their characteristic amoeboid morphology, with absent cytoplasmic projections (acanthopodia), appearing instead as rounded and irregular cells. Although these cells were not undergoing visible degranulation, they displayed signs of sensitization and possible functional impairment, likely due to the action of chlorhexidine, C_16_MImCl or the commercial solution (Fig. 2C). These trophozoites were also excluded from adhesion counts.

**Table 2:** Antiamoebic activity of IS against acanthamoeba spp. Trophozoites adhered to contact lenses

Isolate MZ404337		Isolate MZ404332	
Condition	Amoeba adhesion	Condition	Amoeba adhesion
NC	Yes	NC	Yes
PC	No	PC	No
IS [156.2 µg/mL]	No	IS [78.1 µg/mL]	No
Commercial solution	Yes	Commercial solution	Yes

NC: negative control; PC: positive control; IS: imidazolium salt

The results confirming amoeba viability through CL inoculation on NNA plates are presented in Table 3.

**Table 3:** Confirmation of acanthamoeba viability via CL inoculation on NNA plates

Isolate MZ404337		Isolate MZ404332	
Condition	Amoeba growth	Condition	Amoeba growth
NC	Yes	NC	Yes
PC	No	PC	No
IS [156.2 µg/mL]	No	IS [78.1 µg/mL]	No
Commercial solution	Yes	Commercial solution	Yes

NC: negative control; PC: positive control; IS: imidazolium salt

Data from Tables 2 and 3 demonstrated that the IS rendered 100% of the trophozoites nonviable in both isolates. In contrast, even though some sensitization occurred, the commercial solution only rendered part of the adhered trophozoites unviable, allowing their subsequent growth on NNA plates. Chlorhexidine also rendered all trophozoites unviable.

In addition, HPLC analysis of the assay medium after CL removal revealed no detectable levels of C_16_MImCl above the instrument’s detection limit, even at the initial concentrations of 156.2 µg/mL (isolate MZ404337) and 78.1 µg/mL (isolate MZ404332).

### HCE-T Cell Viability Assay

**Fig. 3:**
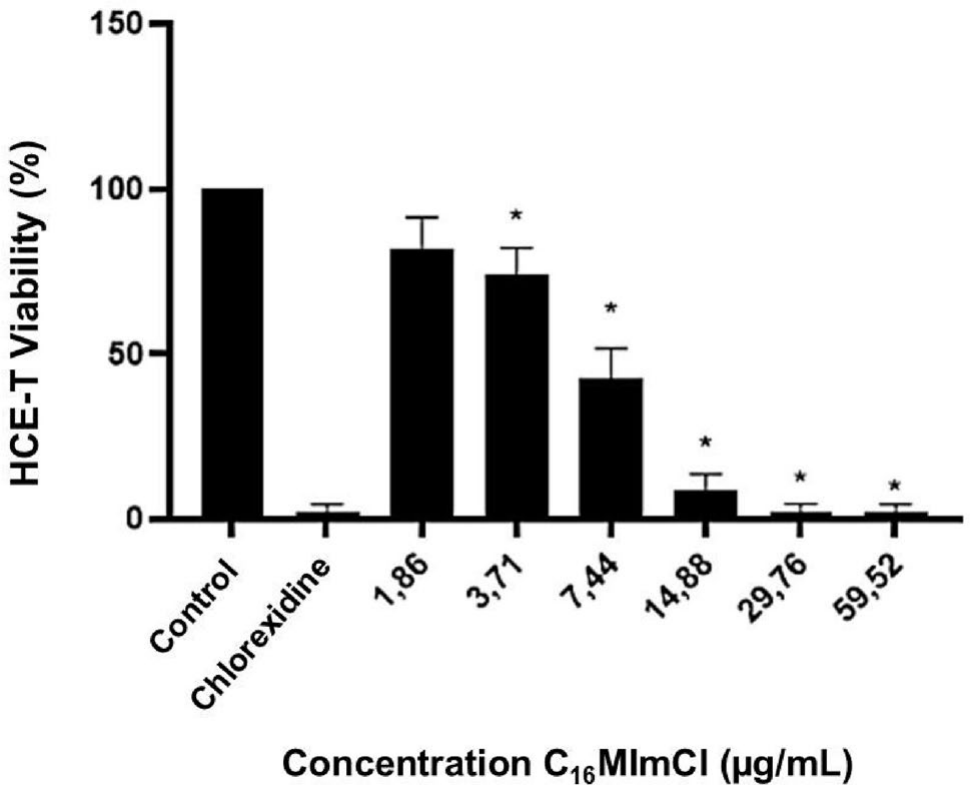
HCE-T cell viability after 2 h of exposure to the IS C_16_MImCl, expressed as percentage of viable cells. *Significant differences compared to the negative control are indicated (*p* < 0.001). The negative control refers to untreated cells, while the positive control corresponds to treatment with 0.02% chlorhexidine

Fluorescence data revealed that after 2 h of incubation, cell viability at 1.86 µg/mL C_16_MImCl was comparable to the untreated control. As the concentration increased, a dose-dependent decrease in viability was observed. From 14.88 µg/mL onward, cell viability approached levels similar to those observed with 0.02% chlorhexidine (Fig. 3).

**Fig. 4:**
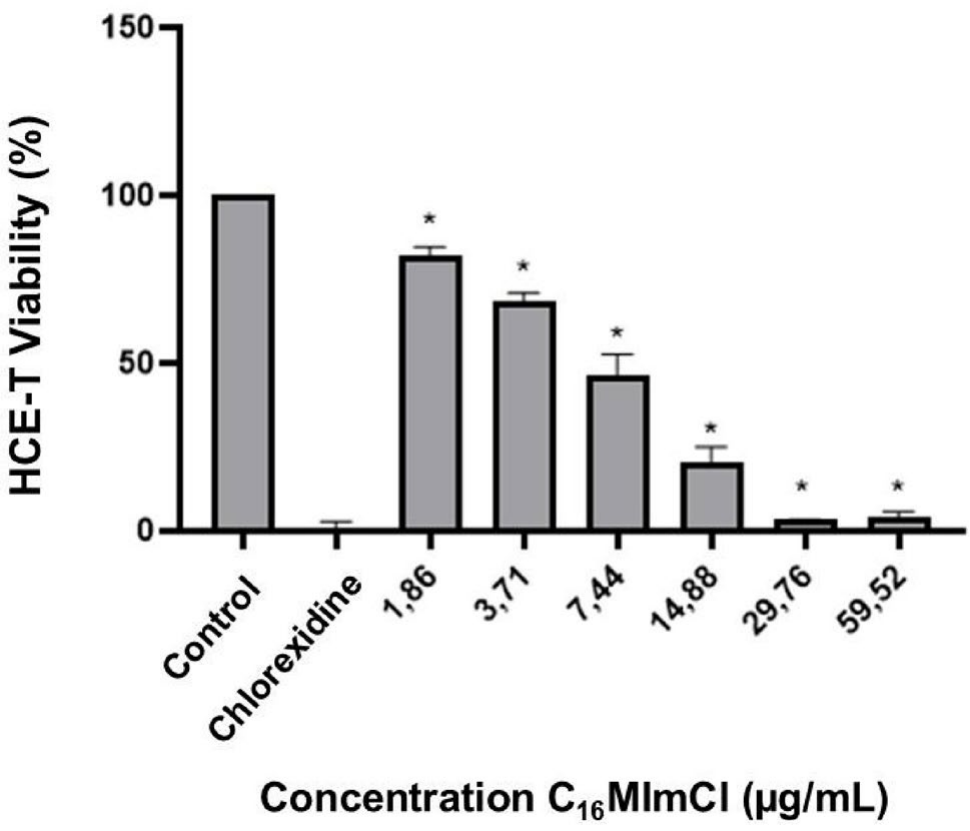
Viability of HCE-T cells after 4 h of exposure to the IS C_16_MImCl, expressed as percentage of cell viability. *Significant difference compared to the negative control (*p* < 0.001). The negative control refers to untreated cells; the positive control corresponds to 0.02% chlorhexidine

After 4 h of incubation with C_16_MImCl, HCE-T cell viability showed a similar dose-dependent decline as observed in the 2-hour assay. From 29.76 µg/mL onward, viability levels approached those observed with 0.02% chlorhexidine (Fig. 4).

The statistical test applied, One Way Anova, is an essential method used to determine whether there are significant differences among three or more means obtained from an experiment, analyzing the influence of only one factor [43].

## Discussion

CL can act as vectors for amoeba adhesion [44]. The type of material, surface roughness, and water content of the CL influence the adhesion of *Acanthamoeba* trophozoites through their acanthopodia, which are spiny projections on the surface of the trophozoites [45–49]. In addition, the formation of bacterial biofilms also contributes to increased trophozoite adhesion [50].

Due to the substantially higher adhesion of *Acanthamoeba* spp. trophozoites to CL compared to cysts [51, 52], and because trophozoites represent the metabolically active and infective form of *Acanthamoeba* spp. [53], the present study primarily aimed to quantify the efficacy of C_16_MImCl against trophozoites only. A previous study by our group [36] demonstrated the efficacy of C_16_MImCl against *Acanthamoeba* spp. cysts, determining the 24-hour MIC and showing that 100% of cysts were inactivated at concentrations of 7.81 µg/mL (isolate MZ404337) and 1.95 µg/mL (isolate MZ404332). However, that study evaluated the activity of C_16_MImCl under experimental conditions that did not include CL.

Improper cleaning of CL contributes to *Acanthamoeba* contamination [9]. Initially, trophozoites adhere to the CLs; then, they are transferred to the eyes, and if there is any microlesion, adhesion occurs, followed by penetration into the corneal epithelium. A series of processes take place from this point, but in summary, corneal tissue degradation occurs, ultimately leading to AK [9, 48]. Therefore, studying adhesion models of *Acanthamoeba* is clinically relevant because adhesion to corneal epithelial cells is the first step in infection, and understanding this process can help develop better disinfectants and therapies.

Current multipurpose solutions are unable to completely eliminate adhered trophozoites [41] and may induce encystment [54]. This is significant because cysts are more resistant to treatments, and once in contact with the eyes, these cysts can revert to the trophozoite form and invade corneal tissue [19, 55].

This study initially aimed to evaluate the efficacy of C_16_MImCl at the 24-hour MIC previously established for *Acanthamoeba* spp. isolates [35]. However, preliminary tests revealed that these concentrations were insufficient to inactivate trophozoites when applied to CL, as viable growth was observed on NNA plates (data not shown). This reduction in efficacy is likely due to an interaction between the lens material and the IS, resulting in partial absorption of the compound and reducing the active concentration available to act on the amoebae.

To investigate the possible interference of CL in the activity of the IS and whether, by chance, a change in the IS batch might have altered its action, an experiment was conducted with isolate MZ404332. The MIC was tested without the presence of CL, with each replicate performed with IS from different batches, one from year 2023 and one from year 2025. The results obtained through visualization with Trypan blue indicated the nonviability of all trophozoites after 24 h in contact with the IS from both batches, and these findings were confirmed by the absence of trophozoite growth on the NNA plates. Therefore, the IS C_16_MImCl maintained its amoebicidal activity even after being stored for different periods, as it presents properties such as high thermal and chemical stability, in addition to being non-volatile [31, 56].

The pilot test consisted of increasing the IS MIC by 5-fold and 10-fold within 24 h. The results indicated that a concentration 5-fold higher than the MIC was not sufficient for complete elimination of trophozoites. Therefore, a concentration 10-fold higher than the MIC was established for definitive testing with both isolates.

In previous work [36, 37], the MIC for C_16_MImCl were 15.62 µg/mL (MZ404337) and 7.81 µg/mL (MZ404332). In the current study, concentrations ten times higher were required to achieve amoebicidal activity (156.2 µg/mL and 78.1 µg/mL, respectively), confirming the hypothesis that the lens material interferes with drug availability.

Soft CL composed of silicone hydrogel are designed to facilitate oxygen permeability and absorb water [57]. Saez-Martinez et al. [58] demonstrated, through Environmental Scanning Electron Microscopy (ESEM), that the silicone hydrogel Comfilcon A has high ion permeability due to a high porosity in the CL structure. The hydrogels form a network of polymers (pores) that retain small molecules, drugs and ions that are in their aqueous form [58–60]. Silicone hydrogel CL absorbs aqueous solutions better [61].

Consequently, CL with a higher water content (hydrophilic nature) have a greater capacity to absorb water-soluble drugs and can release them, for example, through tears. Therefore, CL made with this type of material are the most recommended for therapeutic use [59]. Their physicochemical properties—such as porosity, water theory, and absence of significant ionic charge—allow the passive absorption of small, water-soluble, and lipophilic molecules [62]. Therefore, the interaction of the CL with C_16_MImCl is possibly controlled by three mechanisms: polymer porosity, hydrophilicity, and ionic interactions. Based on these properties, it can be inferred that CL composed of Comfilcon A can retain small amounts of IS (aqueous solution). Although the solutions tested—C_16_MImCl, chlorhexidine, and the commercial multipurpose solution—are all cationic, the lenses used are non-ionic [63], minimizing electrostatic interactions but still allowing possible retention by acquisition in the pores.

The adhesion of trophozoites to CLs made of Comfilcon A is lower than to other materials, as their surface is smoother [47]. The CL used in this study were new and had not been previously worn, which may have contributed to the reduced adhesion of the amoebae [64]; nevertheless, trophozoite adhesion to these CL was still observed.

The evaluation of *Acanthamoeba* spp. trophozoite adhesion to CL was performed qualitatively, indicating only the presence or absence of adhesion. This approach is justified, as the adhesion of a single trophozoite is sufficient to represent an infection risk, making numerical quantification less relevant for the objectives of this study. Therefore, infection risk was assessed without considering the magnitude of the parasitic load. When reported as absence of adhesion, it indicates that no trophozoites were detected after examining the entire surface of the CL under a microscope. Scanning electron microscopy is planned for observing trophozoites adhered to CL; however, due to the project’s time constraints, this analysis could not be performed and is considered a future perspective.

Consistent with these properties, the HPLC data confirmed that no residual C_16_MImCl was detected in the assay medium after lens removal. This suggests a depletion below the detection limit, which may indicate adsorption/partitioning, binding to the container, or degradation; however, the experiment does not allow determination of which of these mechanisms occurred. This result supports the hypothesis that the compound interacts strongly with the lens material and was largely retained or absorbed. This likely retention provides a mechanistic explanation for the reduced efficacy observed at the previously established MIC values, since the active compound available in solution was possibly diminished by interaction with the CL.

This interaction may explain the observed reduction in amoebicidal efficacy, as a portion of the IS likely adhered to or was retained by the lenses. However, at the higher concentrations used, trophozoites exhibited morphological signs of cell lysis and did not grow on NNA plates, indicating successful inactivation. Importantly, C_16_MImCl did not induce encystment likely due to its rapid trophozoite-killing action and because, according to Dos Santos et al. [65], the inactivation process mediated by C_16_MImCl is continuous—unlike some commercial solutions that have been shown to trigger immature cyst formation [28]. The damage caused by C_16_MImCl to amoebic cells, at amoebicidal concentrations, is irreversible, preventing trophozoites from encysting by blocking the activation of stress-induced encystment pathways [66]. In contrast, for encystment to occur, in the case of multipurpose solutions, these solutions must exhibit limited efficacy or slow action, as indicated by the studies of Kilvington et al. and Kovacs et al. [28, 67].

Most CL cleaning and disinfecting solutions were developed to eliminate bacteria and protein and lipid residues, and not specifically to target *Acanthamoeba* spp [55]. The cleaning and disinfection solution selected for this study is widely used on the market and representative of real-use conditions [68, 69]. The objective was not to compare commercial brands, but to use a reference standard to evaluate the effect of C_16_MImCl. Consistent with previous findings [41, 70], the commercial solution tested in this study failed to consistently eliminate *Acanthamoeba* spp. trophozoites. While some reduction in viability was observed, it was insufficient for complete eradication, and trophozoite adhesion persisted across replicates—likely due to absorption of the active compounds by the lens material.

These results underscore the need for new formulations that account for drug-lens interactions. C_16_MImCl shows promise as an active agent in CL disinfecting solutions, but its presumed absorption by lenses raises concerns about efficacy and potential ocular toxicity. Notably, in cytotoxicity assays, C_16_MImCl was less harmful to corneal cells at lower concentrations than 0.02% chlorhexidine (the positive control), while still demonstrating effective amoebicidal action. Interestingly, a slight increase in cell viability at 14.88 µg/mL after 4 h compared to 2 h suggests a possible cellular stress adaptation response, rather than a true recovery in viability [71, 72].

The amoebicidal concentrations evaluated in this study (78–156 µg/mL, i.e., 0.0078–0.0156% w/v) are higher than the levels of some preservative disinfectants used in multipurpose solutions (e.g., polyquaternium-1 at 0.001% and myristamidopropyl dimethylamine at 0.0005%) [68, 73], but remain well below hydrogen peroxide-based disinfection systems (~ 3% hydrogen peroxide) [73] and also below the concentration of chlorhexidine used for the treatment of *Acanthamoeba* keratitis (0.02%) [74]. The effective concentration against *Acanthamoeba* spp. trophozoites determined in this study are higher than the concentration considered safe for human cells, indicating a negative or narrow therapeutic window under these conditions. However, as the intended application is for CL disinfection and not ocular administration, safety will depend on the persistence of the product after the recommended regimen; therefore, these data should be interpreted as an initial risk screening rather than as evidence of ocular tolerability.

Although C_16_MImCl exhibits some cytotoxicity at higher concentrations, it is active against trophozoites at lower concentrations than 0,02% chlorhexidine (i.e. 200 µg/mL). However, in vitro assays using monolayers do not replicate the complex in vivo ocular environment. Studies have shown that, in in vitro assays, multipurpose solutions can exhibit cytotoxicity to corneal cells [75, 76] due to direct exposure or dilutions that do not accurately reflect the actual clinical response in the human cornea. Under physiological conditions, the solutions are diluted by tears, the action of blinking, and enzymes [77]. Therefore, assays using cell monolayers are generally more sensitive to drugs. Thus, further studies using advanced models—including animal studies—are necessary to confirm its safety and efficacy [78].

Given its potential, C_16_MImCl could be developed as a component of multipurpose CL cleaning and storage solutions. It is not currently proposed for direct ocular application, such as eye drops, due to toxicity concerns. The distinction between eye drops and multipurpose solutions lies in the fact that eye drops are formulated for direct application to the eyes and are considered safe for such use because their composition is compatible with the ocular surface. In contrast, multipurpose solutions are intended for cleaning, disinfecting, rinsing, and storing CL and their storage cases, and are not recommended for direct ocular use because they contain disinfecting agents. Multipurpose solutions are safe when in indirect contact with the eyes (through CL). However, when used improperly and applied directly to the eyes, they may cause irritation, burning, redness, and even damage to ocular tissue. However, mechanisms like tear flow, blinking, and enzymatic activity may reduce IS concentration on the ocular surface over time, mitigating its toxic effects [79].

As a consequence of the increasing number of AK cases, a public health concern has emerged. Since CL use has become increasingly popular, the easy access to lenses—often without proper guidance regarding their use and hygiene—can make CL users more susceptible to developing AK [80, 81]. Currently, the demand for CLs is not limited to correcting visual impairments but also extends to cosmetic purposes, such as colored or cosmetic CL [82, 83]. Studies indicate that this type of CL further promotes amoeba adhesion due to its rougher and more irregular surface, which in turn contributes to bacterial biofilm formation, influencing *Acanthamoeba* adhesion [49]. Both healthcare professionals and CL users should be informed and made aware of the risks associated with improper CL use [82, 84].

Future studies should explore the use of C_16_MImCl in CL cases, assess its activity in shorter exposure times, and evaluate its potential for incorporation into lenses for controlled drug release—providing sustained therapeutic activity for conditions such as amoebic keratitis. Furthermore, the results presented demonstrate the activity of the product in the context of CL care, but they do not establish commercial viability or ocular safety, which will depend on the formulation, lens compatibility, residual transfer, and regulatory toxicology. Therefore, additional studies are still required to more precisely determine the concentration that balances amoebicidal efficacy with ophthalmic safety.

## Conclusion

This study highlights the promising potential of the IS C_16_MImCl as an amoebicidal agent against *Acanthamoeba* spp. trophozoites readily adhered to soft CL, reinforcing the need for effective disinfection strategies. C_16_MImCl prevented the adhesion of trophozoites to CLs, inactivating them more effectively than a commercial multipurpose solution and showed lower cytotoxicity to corneal cells than 0.02% chlorhexidine at lower concentrations. Although higher concentrations were required due to possible interactions with the lens material, these findings support the potential of C_16_MImCl for developing improved cleaning and storage solutions for CL. Future work should focus on optimizing its concentration to ensure efficacy against both trophozoites and cysts while minimizing cytotoxicity.

## Data Availability

No datasets were generated or analysed during the current study.
